# The Yeomanry Hospital

**Published:** 1900-07-14

**Authors:** 


### THE YEOMANRY HOSPITAL.

Messrs. J. Defries and Sons write: We notice that in the article by Dr. Alfred D. Fripp in your issue of the 23rd ult. that a Washington Lyon's disinfector is employed by the Imperial Yeomanry Hospital. We beg to inform you that this statement has been made in error, the disinfector in use being the Equifex Saturated Steam apparatus manufactured by ourselves.

*** We published the article as we received it from Mr. Fripp, to whom we have forwarded a copy of Messrs. Defries' letter.—[Ed. *T. H.*]

